# Clomiphene Citrate in the Management of Infertility in Oligospermic Obese Men with Hypogonadism: Retrospective Pilot Study

**DOI:** 10.3390/medicina59111902

**Published:** 2023-10-26

**Authors:** Manesh Kumar Panner Selvam, Saradha Baskaran, Jacob Tannenbaum, Jacob Greenberg, Hosam Y. Shalaby, Wayne J. G. Hellstrom, Suresh C. Sikka

**Affiliations:** 1 Department of Urology, School of Medicine, Tulane University, New Orleans, LA 70112, USA; saradhabaskaran@gmail.com (S.B.); hshalaby@tulane.edu (H.Y.S.); whellst@tulane.edu (W.J.G.H.); 2 School of Medicine, Tulane University, New Orleans, LA 70112, USA; jtannenbaum@tulane.edu

**Keywords:** obesity, hypogonadism, clomiphene citrate, male infertility, semen parameters

## Abstract

*Background and Objectives:* Obesity is a significant risk factor for hypogonadism and infertility that is further associated with reduced semen quality. The aim of this study is to evaluate the effect of clomiphene citrate (CC), prescribed for treating infertility, on serum testosterone and semen parameters, particularly in oligospermic obese hypogonadal men. *Materials and Methods*: A retrospective analysis of data related to men (*n* = 53) who underwent CC treatment for infertility and hypogonadism (testosterone < 300 ng/dL) was performed. Patients with obesity (BMI ≥ 30 kg/m^2^) and sperm concentration ≤ 15 × 10^6^/mL were included for analysis. *Results*: The overall results showed that, in oligospermic obese men (*n* = 31), treatment with CC significantly improved baseline sperm concentration (4.5 ± 6.8 × 10^6^/mL vs. 11.4 ± 15.5 × 10^6^/mL, *p* < 0.05) and motility (31.5% ± 21.5% vs. 42.6% ± 14.7%, *p* < 0.05). Furthermore, subsequent examination of oligospermic hypogonadal obese men treated with CC (*n* = 13) revealed substantial improvements in baseline serum testosterone levels (193.8 ± 59.3 ng/dL vs. 332.7 ± 114.8 ng/dL, *p* < 0.05) along with an increase in sperm concentration, total motility, and normal morphology. *Conclusions*: The results of this retrospective study suggest that CC treatment not only improves chances of fertility outcomes by substantially improving semen parameters but also increases total serum testosterone levels in oligospermic obese men without any supplemental and expensive testosterone replacement therapy.

## 1. Introduction

Infertility is a global health issue impacting about 8–12% of couples, with the male factor as the sole causative in about two-thirds of these cases [1]. Diagnosis of male infertility typically involves a comprehensive assessment of medical history, physical examination, semen analysis, and possibly further tests such as hormonal evaluations, genetic tests, and imaging studies. Semen analysis is a critical laboratory diagnostic tool used to assess male fertility and to identify potential causes of infertility. A significant decline in semen quality has been observed in the last few decades and is associated with lifestyle factors (such as poor diet, excessive alcohol consumption, tobacco use (smoking), drug use, and obesity) among modernized communities worldwide [2]. When semen quality is compromised, it can contribute to male infertility, which is defined as the inability to conceive after one year of regular, unprotected intercourse.

There has been a parallel increase in the rates of obesity in reproductive-age men in westernized society [3]. Several studies have shown a clear link between obesity and a higher risk of male reproductive issues. Incidence of male fertility is observed to be reduced by approximately 10% for a 10 Kg increase in weight [4]. A meta-analysis revealed that obese men are more likely to have oligospermia or azoospermia than normal-weight men [5]. In addition, obese men experience reduced success rates with assisted reproductive techniques such as in vitro fertilization (IVF) or intracytoplasmic sperm injection (ICSI), and the risk of complications during pregnancy are higher for obese couples.

Obesity affects male fertility by altering the hypothalamic–pituitary–gonadal (HPG) axis, disrupting testicular steroidogenesis and metabolic dysregulation [6,7]. Obesity is associated with reduced semen quality and is a major risk factor for hypogonadism [8]. Low testosterone levels are a hallmark of male hypogonadism. The American Urological Association (AUA) defines male hypogonadism as a state of total testosterone level less than 300 ng/dL combined with low testosterone symptoms such as reduced libido, reduced energy, depression, erectile dysfunction, reduced muscle mass, and fatigue [9]. This testosterone deficiency can occur at any stage of life from fetal development to adulthood, but the incidence of male hypogonadism is more prevalent in older men.

Testosterone replacement therapy (TRT) is the mainstay treatment for symptomatic male hypogonadism and is designed to restore testosterone levels in individuals with low testosterone [10]. Success of TRT depends on appropriate medical evaluation and extent of improvement in the symptoms associated with hypogonadism with low testosterone and how much it improves the overall well-being of affected individuals. However, exogenous testosterone has been known to adversely affect aspects of testicular function, mainly spermatogenesis and steroidogenesis, by suppressing the hypothalamic–pituitary–testis (HPT) axis. This leads to the downregulation of luteinizing hormone (LH) and follicle-stimulating hormone (FSH) by the feedback inhibitory pathways [11]. TRT is generally not recommended for men with borderline low testosterone levels, especially those who are trying to conceive, as it can lead to infertility due to a significant reduction in sperm count. Clomiphene citrate (CC), an estrogen receptor modulator, increases the secretion of LH and FSH by inhibiting the negative effect of estrogen on the HPT axis. LH stimulates the Leydig cells in the testes to produce testosterone, while FSH supports spermatogenesis by promoting the development of sperm in the testes [12].

CC is also recommended by the AUA in the management of testosterone deficiency [9] and has been reported to increase testosterone levels in male obesity-related hypogonadism [13]. CC treatment improves testosterone levels without having any negative impact on spermatogenesis unlike TRT [14]. However, there is a lack of information on the impact of CC treatment on sperm parameters, especially in obesity-related hypogonadism. The present study aims to retrospectively evaluate the effect of CC treatment on serum testosterone and semen parameters, particularly in oligospermic obese men with hypogonadism in comparison to non-obese men.

## 2. Materials and Methods

### 2.1. Study Design and Ethics Statement

After approval by our institutional review board (IRB #2020-1192), we conducted this retrospective study including 10 years of data on patients with infertility (*n* = 532) obtained from our urology/andrology clinics. Semen samples were collected from these patients with a minimal abstinence duration of 48 h and subjected to evaluation in our CLIA (Clinical Laboratory Improvement Amendments)-certified andrology laboratory for all major sperm parameters within one hour of collection as per WHO guidelines. Both macroscopic and microscopic assessments were carried out after the complete liquefaction of each sample. Hyperviscous samples and those with some coagulum were then gently passed through a 21G needle to make the sample more homogenous. Both macroscopic (such as color, pH, viscosity, and volume) and microscopic (such as sperm concentration, total motility, and morphology) parameters were recorded in our CLIA-certified semen analysis data form.

### 2.2. Semen Analysis

Semen samples were mixed well by swirling prior to the assessment of sperm concentration and total motility. Briefly, 7 µL of semen aliquot was loaded at a 45° angle into a 20 µm two-chambered standard count slide (Leja, Spectrum Technologies, Healdsburg, CA, USA). Sperm were observed under a phase-contrast microscope fitted with a 5 × 5-boxes ocular grid and calibrated for microscope count factor using 20× magnification. For motility evaluation, at least 200 sperm were counted using the grid across a minimum of eight different microscope fields. For the evaluation of sperm concentration, a well-mixed semen sample was first diluted with a sperm immobilization solution before being loaded into the chamber. Sperm were counted in an entire ocular grid across at least six different fields and concentration was calculated as per our standardized calculations using the dilution factor and microscope count factor. The presence of sperm agglutination, round cells, and white blood cells after proper staining was also examined during microscopic evaluation. Sperm concentration was expressed as 10^6^ sperm/mL and total sperm count as millions (10^6^), whereas total motility was expressed as the percentage (%) of motile sperm. Smears were prepared on a clean frosted slide for evaluation of sperm morphology. Briefly, 7 μL of well-mixed semen aliquot was placed on the opposite end of the frosted area of the slide. A spreader slide was used to spread the semen towards the frosted end of the slide at an angle of 45°, making an even thin smear. The Diff-Quick staining protocol was used to stain spermatozoa that were examined using a 100× objective and a 10× eyepiece and a bright field for morphology evaluation according to WHO (1992) criteria with >30% normal head as the cut-off value.

### 2.3. Study Groups

Further, this study also included infertile men (*n* = 80) who were treated with a starting daily dose of 25 mg CC for at least 3 months. Patients were categorized into the following groups based on the inclusion criteria: (A) oligospermic obese (*n* = 31, concentration < 15 × 10^6^ sperm/mL, testosterone > 300 ng/mL, body mass index (BMI) ≥ 30); (B) oligospermic non-obese (*n* = 22, concentration < 15 × 10^6^ sperm/mL, testosterone > 300 ng/mL, BMI < 30); (C) oligospermic hypogonadal obese (*n* = 13, concentration < 15 × 10^6^ sperm/mL, testosterone ≤ 300 ng/mL, BMI ≥ 30); and (D) oligospermic non-obese (*n* = 10, concentration < 15 × 10^6^ sperm/mL, testosterone ≤ 300 ng/mL, BMI < 30). Men with azoospermia and necrozoospermia were excluded from the present study. Patients with genetic defects, urinary tract infections, inflammation of the reproductive tract, and sexually transmitted diseases were excluded from this study. In addition, patients with a history of radiation or chemotherapy were excluded from the analysis.

### 2.4. Statistical Analysis

Baseline demographic data (age and BMI), pre-treatment testosterone levels, and semen parameters (concentration, total motility, and morphology) were evaluated as per WHO guidelines and collected retrospectively. Post-CC treatment data (after 3 months) were used to evaluate the change in testosterone levels and semen parameters compared to those of pre-treatment. Statistical analysis was performed using MedCalc statistical software version 19.1 (MedCalcSoftware bv, Ostend, Belgium). All the variables were tested for normal distribution, and either parametric (paired-samples *t*-test) or non-parametric (Wilcoxon test) tests were employed. Statistical significance was set at a two-tailed *p*-value of <0.05.

## 3. Results

Overall, 53 patients were treated with CC and met the study inclusion criteria. The demographic characteristics of these patients are presented in Table 1. The average BMI values of the obese (*n* = 31) and non-obese (*n* = 22) patients were 37.3 ± 1.2 kg/m^2^ and 26.3 ± 0.6 kg/m^2^, respectively. There was no significant age difference between the two groups. A majority of the patients included in our study were Caucasian (56.6%) followed by African American (35.8%) and Hispanic (7.5%) men. Baseline semen parameters (concentration, total sperm count, total motility, and normal head forms) and testosterone levels are presented in Table 1. Sperm concentration, total motility, and normal head forms were abnormal, whereas white blood cell concentration was in the normal range according to WHO guidelines. The testosterone levels in the non-obese group were normal, whereas, in the obese group, these were slightly above (borderline) the cut-off value (330 ng/dL).

### 3.1. Effect of CC on Semen Parameters and Testosterone Levels in Oligozoospermic Obese Men

The overall results of this study showed that CC treatment significantly improved baseline sperm parameters in oligozoospermic obese men. There was a significant (*p* < 0.05) increase in the average sperm concentration (6.9 ± 13.2 × 10^6^/mL), total sperm count (17.7 ± 41.9 × 10^6^), and total motility (11.1% ± 22.0%) post-treatment (Table 2). No such significant change was noticed in sperm morphology (4.2% ± 12.3%) or serum testosterone levels (55.1 ± 252.3 ng/dL) post-treatment with CC (Table 2).

### 3.2. Effect of CC on Semen Parameters and Testosterone Levels in Oligozoospermic Hypogonadal Obese Men

**Figure 1:**
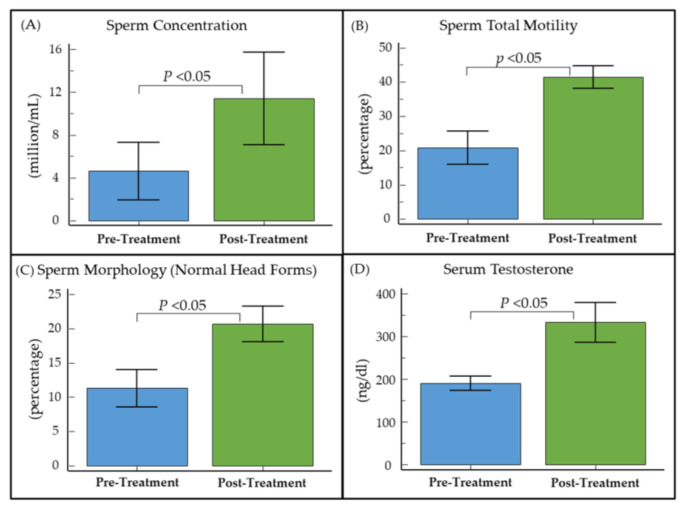
Pre- and post-treatment (**A**) sperm concentration, (**B**) total motility, (**C**) sperm morphology, and (**D**) serum total testosterone levels in oligozoospermic hypogonadal obese men treated with clomiphene citrate for male infertility.

Furthermore, a sub-analysis of oligospermic obese men (*n* = 13) with hypogonadism (serum total testosterone ≤ 300 ng/dL) revealed significant (*p* < 0.05) improvements in baseline sperm concentration (6.8 ± 10.3 × 10^6^/mL), total motility (20.6% ± 22.8%), sperm morphology (9.4% ± 13.8%), and serum testosterone levels (138.8 ± 127.8 ng/dL) (Figure 1 and Table S1).

## 4. Discussion

Obesity has significant negative effects on various aspects of reproductive health, including semen parameters and the risk of hypogonadism [7]. Several studies suggest that obese men show decreased sperm count values [5,15,16]. Elevated BMI and increased body fat contribute to hormonal imbalances that affect sperm production in the testes [17]. Further, obesity is associated with increased levels of certain hormones such as estrogen and insulin [18,19], which lead to the condition known as estrogen dominance or insulin resistance. Alterations in estrogen and insulin levels interfere with steroidogenesis in the testes and affect the normal production of testosterone, leading to hypogonadism. CC is a common medication used to treat infertility in men. Our results showed that, in oligospermic obese infertile men, treatment with CC significantly improved baseline sperm concentration and motility. Furthermore, very interestingly, we observed that oligospermic obese men who were also hypogonadal revealed substantial improvements not only in baseline serum testosterone levels but also in sperm concentration, total motility, and normal morphology when similarly treated with CC.

Obesity is often also characterized by chronic low-grade inflammation throughout the body, which likely affects testicular function, responsible for producing testosterone and sperm [20]. Inflammation in the testes impairs the process of spermatogenesis. Such inflammatory reactions stimulate the production of reactive oxygen species, resulting in oxidative stress, which can damage sperm DNA and negatively affect sperm function and fertilization capability. In addition, the increased production of pro-inflammatory cytokines released during inflammation negatively impacts the production and regulation of sex hormones such as testosterone. Furthermore, hormones linked to obesity, such as insulin and leptin, disrupt the function of the HPG axis, which controls the release of hormones involved in reproductive function [21,22]. In the current retrospective study, we observed lower testosterone levels in obese men compared to non-obese men with poor sperm concentration and other semen parameters. However, treatment with CC improved the testosterone levels in obese men with poor sperm concentration. This was in accordance with previous studies that reported improvements in semen parameters post-CC treatment [23,24,25]. CC is a selective estrogen receptor modulator (SERM) and blocks the negative feedback loop that estrogen usually exerts on the HPG axis, which in turn stimulates the release of hormones, such as LH and FSH, from the pituitary gland [26]. These hormones are crucial in signaling the testes to produce more testosterone [27]. The rise in FSH levels due to CC treatment also supports the process of spermatogenesis, which is particularly beneficial for men with conditions like low sperm count or oligospermia. The results of our current study corroborate the previous findings that CC improves sperm parameters such as concentration, total sperm count, and total motility in oligozoospermic obese men.

Obesity-related hypogonadism has an adverse impact on reproductive function. Many reports suggest that, in obese men, most of the androgens are converted into estrogens, leading to a hormonal imbalance. Secondary hypogonadism specifically involves a dysfunction of the HPG axis, which regulates the production of hormones that stimulate the testes [7]. Inflammatory cytokines and adipokines produced by fat tissue influence the HPG axis, potentially reducing testosterone production. CC is an anti-estrogenic drug that competes with estrogen upon binding to estrogen receptors in the hypothalamus. CC is used as a medication for treating obesity-related hypogonadism to boost the testosterone levels by increasing LH and FSH levels [27,28]. Further, CC is also used as a treatment option for men with oligospermia to improve their semen parameters [25]. When examining the effects of CC on these patients, in particular, oligospermic obese men with hypogonadism (serum total testosterone ≤ 300 ng/dL) had a more pronounced response to the treatment with a significant increase of over 135 ng/dL in serum total testosterone levels as well as a marked improvement in sperm concentration, total motility, and normal morphology. It is important to highlight that, in addition to sperm concentration, total motility and normal morphology are predictors of sperm’s ability to successfully fertilize an ovum under in vitro fertilization or assisted reproductive treatment. Our results suggest that along with improvements in semen parameters, CC is a potentially useful therapeutic option for obese men with oligospermia and associated hypogonadism.

The current retrospective analysis has some limitations. This is a pilot study with a limited number of subjects (*n* = 53) included for statistical analysis. In addition, we have not included other reproductive hormones in our study cohort such as estrogen, FSH, and LH for analysis.

## 5. Conclusions

The findings of this study have implications for the management of oligospermic obese infertile men with hypogonadism. Given the potential benefits of CC treatment, it would be beneficial to further investigate the effect of this treatment in larger cohorts of oligospermic men with obesity to confirm the findings of this retrospective pilot study. Further research should also focus on understanding the underlying mechanisms of the observed effects of CC on testosterone and semen parameters. In summary, our study very interestingly suggests that CC treatment not only improves chances of fertility outcomes by substantially improving sperm parameters but also increases serum testosterone levels in oligospermic obese men without any supplemental and expensive testosterone replacement therapy.

## Figures and Tables

**Table 1:** Baseline overall patient characteristics (*n* = 53).

Patient Characteristics	
***Age (years)***	
Obese group (*n* = 31)	36.9 ± 1.5
Non-obese group (*n* = 22)	34.0 ± 1.2
***Body Mass Index (kg/m^2^)***	
Obese group (*n* = 31)	37.3 ± 1.2
Non-obese group (*n* = 22)	26.3 ± 0.6
***Total Testosterone (ng/dL)***	
Obese group (*n* = 26)	304.9 ± 31.6
Non-obese group (*n* = 22)	369.6 ± 42.9
***Race***	
White	30 (56.6%)
African American	19 (35.8%)
Hispanic	4 (7.5%)
***Semen parameters***	
Semen volume (mL)	2.9 ± 0.2
pH	7.6 ± 0.1
Sperm concentration (×10^6^/mL)	3.7 ± 0.8
Total sperm count (×10^6^)	11.9 ± 4.2
Total motility (%)	30.3 ± 2.8
Sperm morphology (normal heads) (%)	14.8 ± 1.4
White blood cells (WBC) (×10^6^/mL)	0.9 ± 0.2

Note: Values reported as mean ± SEM or number (per cent cohort).

**Table 2:** Pre- and post-treatment semen parameters and testosterone levels in oligozoospermic obese men treated with clomiphene citrate for male infertility.

Parameters	Pre-Treatment	Post-Treatment	*p*-Value
	Mean ± SD	Mean ± SD	
Sperm Concentration (×10^6^/mL)	4.5 ± 6.8	11.4 ± 15.5	<0.05
Total Sperm Count (×10^6^)	13.8 ± 38.5	31.5 ± 43.9	<0.05
Motility (%)	31.5 ± 21.5	42.6 ± 14.7	<0.05
Normal Head Forms (%)	16.1 ± 10.9	20.3 ± 9.0	0.07
Testosterone (ng/dL)	349.8 ± 174.5	404.9 ± 157.9	0.09

Note: *p*-value < 0.05 is considered statistically significant.

## Data Availability

Data of our study are available from the corresponding author upon reasonable request.

## Supplementary Materials

The following supporting information can be downloaded at: https://www.mdpi.com/article/10.3390/medicina59111902/s1, Table S1: Pre- and post-treatment semen parameters and testosterone levels in oligozoospermic hypogonadal obese men treated with clomiphene citrate for male infertility.

## References

[1] Vander Borght M., Wyns C. (2018). Fertility and infertility: Definition and epidemiology. Clin. Biochem..

[2] Levine H., Jørgensen N., Martino-Andrade A., Mendiola J., Weksler-Derri D., Mindlis I., Pinotti R., Swan S.H. (2017). Temporal trends in sperm count: A systematic review and meta-regression analysis. Hum. Reprod. Update.

[3] Dixon T., Waters A.-M., Dixon T. (2003). A Growing Problem: Trends and Patterns in Overweight and Obesity among Adults in Australia, 1980 to 2001.

[4] Sallmén M., Sandler D.P., Hoppin J.A., Blair A., Baird D.D. (2006). Reduced fertility among overweight and obese men. Epidemiology.

[5] Sermondade N., Faure C., Fezeu L., Lévy R., Czernichow S. (2012). Obesity and increased risk for oligozoospermia and azoospermia. Arch. Intern. Med..

[6] Katib A. (2015). Mechanisms linking obesity to male infertility. Cent. Eur. J. Urol..

[7] Leisegang K., Sengupta P., Agarwal A., Henkel R. (2021). Obesity and male infertility: Mechanisms and management. Andrologia.

[8] Fernandez C.J., Chacko E.C., Pappachan J.M. (2019). Male Obesity-related Secondary Hypogonadism—Pathophysiology, Clinical Implications and Management. Eur. Endocrinol..

[9] Mulhall J.P., Trost L.W., Brannigan R.E., Kurtz E.G., Redmon J.B., Chiles K.A., Lightner D.J., Miner M.M., Murad M.H., Nelson C.J. (2018). Evaluation and Management of Testosterone Deficiency: AUA Guideline. J. Urol..

[10] Bhasin S., Brito J.P., Cunningham G.R., Hayes F.J., Hodis H.N., Matsumoto A.M., Snyder P.J., Swerdloff R.S., Wu F.C., Yialamas M.A. (2018). Testosterone Therapy in Men With Hypogonadism: An Endocrine Society Clinical Practice Guideline. J. Clin. Endocrinol. Metab..

[11] MacIndoe J.H., Perry P.J., Yates W.R., Holman T.L., Ellingrod V.L., Scott S.D. (1997). Testosterone suppression of the HPT axis. J. Investig. Med..

[12] Wheeler K.M., Sharma D., Kavoussi P.K., Smith R.P., Costabile R. (2019). Clomiphene Citrate for the Treatment of Hypogonadism. Sex. Med. Rev..

[13] Soares A.H., Horie N.C., Chiang L.A.P., Caramelli B., Matheus M.G., Campos A.H., Marti L.C., Rocha F.A., Mancini M.C., Costa E.M.F. (2018). Effects of clomiphene citrate on male obesity-associated hypogonadism: A randomized, double-blind, placebo-controlled study. Int. J. Obes. (Lond.).

[14] Thomas J., Suarez Arbelaez M.C., Narasimman M., Weber A.R., Blachman-Braun R., White J.T., Ledesma B., Ghomeshi A., Jara-Palacios M.A., Ramasamy R. (2023). Efficacy of Clomiphene Citrate Versus Enclomiphene Citrate for Male Infertility Treatment: A Retrospective Study. Cureus.

[15] Ma J., Wu L., Zhou Y., Zhang H., Xiong C., Peng Z., Bao W., Meng T., Liu Y. (2019). Association between BMI and semen quality: An observational study of 3966 sperm donors. Hum. Reprod..

[16] Sekhavat L., Moein M.R. (2010). The effect of male body mass index on sperm parameters. Aging Male.

[17] Palmer N.O., Bakos H.W., Fullston T., Lane M. (2012). Impact of obesity on male fertility, sperm function and molecular composition. Spermatogenesis.

[18] Grossmann M., Thomas M.C., Panagiotopoulos S., Sharpe K., Macisaac R.J., Clarke S., Zajac J.D., Jerums G. (2008). Low testosterone levels are common and associated with insulin resistance in men with diabetes. J. Clin. Endocrinol. Metab..

[19] Stárka L., Hill M., Pospíšilová H., Dušková M. (2020). Estradiol, obesity and hypogonadism. Physiol. Res..

[20] Khanna D., Khanna S., Khanna P., Kahar P., Patel B.M. (2022). Obesity: A Chronic Low-Grade Inflammation and Its Markers. Cureus.

[21] Chan J.L., Mantzoros C.S. (2001). Leptin and the hypothalamic-pituitary regulation of the gonadotropin-gonadal axis. Pituitary.

[22] Janssen J. (2022). New Insights into the Role of Insulin and Hypothalamic-Pituitary-Adrenal (HPA) Axis in the Metabolic Syndrome. Int. J. Mol. Sci..

[23] Sharma D., Zillioux J., Khourdaji I., Reines K., Wheeler K., Costabile R., Kavoussi P., Smith R. (2019). Improvements in semen parameters in men treated with clomiphene citrate—A retrospective analysis. Andrologia.

[24] Jiang T., Sigalos J.T., Osadchiy V., Santamaria A., Zheng M.H., Modiri N., Regets K.V., Mills J.N., Eleswarapu S.V. (2023). Temporal Changes of Clomiphene on Testosterone Levels and Semen Parameters in Subfertile Men. World J. Men’s Health.

[25] Huijben M., Huijsmans R.L.N., Lock M., de Kemp V.F., de Kort L.M.O., van Breda J. (2023). Clomiphene citrate for male infertility: A systematic review and meta-analysis. Andrology.

[26] Bendre S.V., Murray P.J., Basaria S. (2015). Clomiphene Citrate Effectively Increases Testosterone in Obese, Young, Hypogonadal Men. Reprod. Syst. Sex. Disord..

[27] Huijben M., Lock M., de Kemp V.F., Beck J.J.H., De Kort L.M.O., van Breda H.M.K. (2023). Clomiphene citrate: A potential alternative for testosterone therapy in hypogonadal males. Endocrinol. Diabetes Metab..

[28] Herzog B.J., Nguyen H.M.T., Soubra A., Hellstrom W.J. (2020). Clomiphene citrate for male hypogonadism and infertility: An updated review. Androg. Clin. Res. Ther..

