# Supplementary material for: Clomiphene Citrate in the Management of Infertility in Oligospermic Obese Men with Hypogonadism: Retrospective Pilot Study
Source: Medicina (Kaunas). 2023 Oct 26;59(11):1902. doi: 10.3390/medicina59111902 (PMC10673313; doi:10.3390/medicina59111902)
Supplement: Supplementary file 1 [file medicina-59-01902-s001.zip › Table S1.pdf]

Table S1: Pre- and post-treatment semen parameters and testosterone levels in oligozoospermic hypogonadal obese men treated with clomiphene citrate for male infertility

Parameters	Pre-Rx	Post-Rx	Difference	p-value
	**Mean ± SD**	**Mean ± SD**	**Mean ± SD**	
Sperm Concentration ($x10^6$/mL)	4.7 ± 9.8	11.4 ± 15.6	6.8 ± 10.3	<0.05
Total Sperm Count ($x10^6$)	20.7 ± 59.5	42.9 ± 63.4	22.1 ± 62.1	0.22
Motility (%)	20.8 ± 17.3	41.5 ± 11.8	20.6 ± 22.8	<0.05
Normal Head Forms (%)	11.3 ± 9.8	20.7 ± 9.3	9.4 ± 13.8	<0.05
Testosterone (ng/dL)	193.8 ± 59.3	332.7 ± 114.8	138.8 ± 127.8	<0.05

Note: p-value <0.05 is considered statistically significant
